# Midwifery Management of Hiesho in Pregnant Women: A Nationwide Survey in Japan

**DOI:** 10.7759/cureus.89852

**Published:** 2025-08-12

**Authors:** Momoko Kondo, Yoko Asaka

**Affiliations:** 1 Division of Nursing, Graduate School of Medicine, Mie University, Tsu, JPN

**Keywords:** cold sensitivity, complementary and alternative medicine (cam), hiesho, midwifery care, pregnancy

## Abstract

Objective

The symptom of cold extremities, known in Japan as Hiesho, may be associated with risks of complications during pregnancy and delivery. Thus, Hiesho care is clinically important for improving comfort and for preventing potential complications. The purpose of this study is to describe the actual practices of Hiesho care provided to pregnant women by midwives working in hospitals, clinics, and maternity homes across Japan.

Methods

This study employed a descriptive cross-sectional survey design. A questionnaire survey was conducted from January to April 2021, targeting midwives working at obstetric facilities nationwide. The survey included questions on the effect of Hiesho on pregnancy, which pregnant women were considered to require Hiesho care, and the specific care methods used. Chi-square tests were conducted to analyze the response rates from hospitals, clinics, and maternity homes. Free-text responses were categorized based on similarities and differences.

Results

A total of 1,677 midwives responded (response rate: 58.8%). Also, 1,494 midwives (89.1%) reported having experiences providing health guidance, and 1,136 midwives (67.7%) reported having experiences providing physical care as Hiesho care. Compared to their counterparts at hospitals and clinics, midwives at maternity homes were significantly more likely to possess experience in both health guidance (p* *< 0.001) and physical care (p* *< 0.001). Impaired maternal circulation was the most frequently reported health effect of Hiesho in pregnant women (1,535 midwives, 91.5%), with no significant variation across facility types. Among pregnant women identified as needing health guidance or physical care, the most frequently cited reason for providing health guidance was "wearing light clothing" (reported by 973 midwives, 65.5%), whereas the most common reason for providing physical care was symptoms of Hiesho (reported by 830 midwives, 73.5%). No significant differences were found in these responses across different types of facilities.

Regardless of facility type, the most common item of health guidance was wearing socks (1,313 midwives, 87.9%; p = 0.705). Other items of guidance were significantly more common among midwives at maternity homes, who also reported providing a greater number of health guidance items. Regarding physical care, the most frequently used method was foot bath (971 midwives, 85.8%), with no significant differences in response rates between facilities (p = 0.772). Moxibustion and Thermie therapy were performed by 150 midwives (13.3%) and 58 midwives (5.1%), respectively, with significantly higher response rates among midwives at maternity homes (moxibustion: p < 0.001; Thermie therapy: p < 0.001).

Conclusion

A total of 1,494 midwives (89.1%) reported having experience providing health guidance as Hiesho care, and 1,136 midwives (67.7%) reported having experience providing treatment. Significantly more midwives working in maternity homes had experience with both health guidance and physical care compared to those working in hospitals and clinics. Midwives recognized that Hiesho negatively affects maternal circulation in pregnant women. The midwives working in maternity homes frequently implemented Hiesho care and often incorporated complementary and alternative medicine (CAM) into their practice. Further research is needed to examine the evidence and effectiveness of the Hiesho care practices that have been implemented thus far.

## Introduction

The symptom of cold extremities, known in Japan as Hiesho, is more common in women than men and often begins in adolescence [1]. Therefore, pregnant women of reproductive age are more likely to experience Hiesho. This condition results from reduced blood flow, with primary vascular dysregulation (PVD) identified as a potential cause [1]. PVD involves inadequate vasodilation or inappropriate venous expansion, leading to impaired circulation. Dysregulation can occur in any organ and may disrupt the autoregulation of blood flow in the heart, limbs, and eyes. Previous research has suggested that Hiesho may be associated with risks such as preterm birth, premature rupture of membranes, prolonged labor, and intrapartum hemorrhage, as well as gestational hypertension [2]. As such, intervention programs targeting Hiesho care for pregnant women have been proposed for midwives and nurses [3,4]. However, the actual Hiesho care practices provided by midwives have not been extensively investigated. In Japan, midwives mainly work in hospitals, clinics, and maternity homes [5]. Each facility supports different levels of pregnancy and delivery complications among pregnant women. Therefore, differences in the experience level of midwives across facilities can influence the type of care provided. Clarifying differences in Hiesho care among Japanese midwives facilitates the sharing of practical methodologies. Furthermore, this can contribute to studies examining the association between Hiesho and pregnancy or delivery complications. The purpose of this study is to clarify the actual practices of Hiesho care provided to pregnant women by midwives working in hospitals, clinics, and maternity homes across Japan.

## Materials and methods

This study employed a descriptive cross-sectional survey design.

Sub-objectives

This study aimed to examine whether midwives’ recognition related to its health impacts differs depending on the type of facility where they are employed (hospitals, clinics, or maternity homes), describe the specific health guidance and physical care practices that midwives currently provide to pregnant women for Hiesho, and explore how the implementation of Hiesho care varies by facility type and the midwives’ perceptions of its effectiveness.

Definition of Hiesho and Hiesho care

Hiesho is "a subjective feeling of coldness in the extremities or body despite a normal core temperature," which is culturally recognized in Japan. Hiesho care is defined as any clinical intervention (direct or educational) aimed at alleviating the symptoms of Hiesho in pregnant women, including health guidance (e.g., lifestyle advice, use of warming garments) and physical care (e.g., moxibustion, foot baths).

Hypothesis of the study

This study was designed based on the hypothesis that both the impact of Hiesho on pregnancy and the implementation of Hiesho-related care vary depending on the type of facility in which the midwife works.

Participants

The participants of this study are midwives working at hospitals, clinics, and maternity homes with obstetric services nationwide. The sample size was estimated using stratified random sampling with the epiR package in R (version 4.3.0; R Foundation for Statistical Computing, Vienna, Austria). To determine the appropriate sample size for each facility type, we referred to the national distribution of midwives across hospitals, clinics, and maternity homes. Based on the 2019 data, there were 22,877 midwives working in hospitals, 9,968 in clinics, and 2,281 in maternity homes [6]. Assuming implementation rates of 60% in hospitals, 80% in clinics, and 90% in maternity homes, a maximum relative error of 0.05, and a confidence level of 95%, the required sample sizes were calculated to be 445 for hospitals, 198 for clinics, and 119 for maternity homes, for a total of 762.

Data collection

Based on the average number of midwives per facility (2.8 for hospitals, 3.2 for clinics, and 0.7 for maternity homes), the required number of facilities was estimated to be 318 hospitals, 124 clinics, and 106 maternity homes. Assuming a 50% response rate, we randomly selected 635 hospitals, 248 clinics, and 211 maternity homes, totaling 1,094 facilities.

The sampling frame consisted of 996 hospitals, 1,215 clinics, and 239 maternity homes listed in official directories published by the Japan Society of Obstetrics and Gynecology and the Japan Midwives Association. Stratified random sampling was conducted based on facility type to ensure proportional representation. Within each stratum, facilities were assigned unique identification numbers and randomly selected using a computer-generated random number table.

Letters requesting participation were sent to the facility managers. Of the 1,094 facilities contacted, 293 agreed to participate (190 hospitals, 31 clinics, and 72 maternity homes). Questionnaires and prepaid return envelopes were distributed to each facility based on the number of midwives employed. Midwives completed the questionnaires anonymously and returned them directly to the researchers. The data collection period was from January to April 2021.

Questionnaire

The questionnaire was developed through consultation with experts in midwifery and maternal nursing, based on midwifery textbooks. The questionnaire development process was as follows: A preliminary framework for the questionnaire was developed based on relevant literature and prior studies, in alignment with the study objectives. Expert opinions were obtained from nine midwives with clinical experience regarding the appropriateness of the question content, selection of response options, and the inclusion of open-ended items. Interview participants included midwives with four to 21 years of clinical experience (mean = 9.3 years, SD = ±5.2), of whom six had experience working at university hospitals, three at general hospitals or clinics, and one each with experience in the Japan International Cooperation Agency (JICA), private practice, and as nursing faculty. A draft questionnaire was then constructed based on insights from the interviews. A pilot test was conducted with clinical midwives and nursing faculty to assess the clarity and relevance of the items, the ease of responding, the time required to complete the questionnaire, and whether the responses aligned with the researchers’ intent. The questionnaire was revised and finalized based on the results of the pilot test. This study conducted an exploratory assessment of internal consistency for three-item sets: the perceived health effects of Hiesho during pregnancy, the content of health guidance, and the types of physical care provided by calculating Cronbach’s alpha coefficients. All items were binary (yes/no), and although Cronbach’s alpha is typically used for continuous variables, it was employed here as a practical indicator to evaluate consistency across items assumed to reflect a common latent construct. The alpha coefficient for the eight items related to the perceived health effects of Hiesho during pregnancy was 0.677, indicating acceptable internal consistency. In contrast, the alpha coefficients for the contents of health guidance (0.592) and the types of physical care provided (0.053) were insufficient, so we did not compute total scores for these domains. Instead, we conducted chi-square tests for each individual item to examine relationships with other variables.

Respondents were asked to select, using multiple responses, the perceived health effects of Hiesho during pregnancy. The list consisted of eight items, including effects such as uterine contractions, fetal developmental concerns, and impaired maternal circulation. The "Other (please specify)" option was also included as a free-text field. In addition, respondents were asked whether they had any experience in providing health guidance or physical care for pregnant women with Hiesho. Those who answered "Yes" to either were presented with a series of follow-up questions. Specifically, respondents with experience in providing health guidance were asked to indicate (1) the types of pregnant women to whom they provided guidance (selected from seven items, with an "Other" free-text field) and (2) the content of health guidance (also selected from seven items, including topics such as clothing, diet, and indoor environment, with an "Other" option). Similarly, respondents with experience in providing physical care were asked to indicate (1) the types of pregnant women for whom they provided care (selected from five items, with an "Other" field) and (2) the types of physical care provided (selected from four items, such as hot compresses, foot baths, moxibustion, and Thermie therapy, with an "Other" option). All items in the questionnaire were dichotomous, with responses recorded as either “yes” (1) or “no” (0). A free-text field was also included to allow participants to describe any other care practices they were currently implementing for Hiesho.

Data analysis

Descriptive statistics were calculated using IBM SPSS Statistics for Windows, Version 27 (Released 2019; IBM Corp., Armonk, New York, United States). For questions that allowed multiple responses, each option was treated as an individual binary variable (1 = selected, 0 = not selected) for analysis. Chi-square tests or Fisher's exact test were used to compare responses across facility types. For significant results, Holm’s method was used for post hoc test. Missing data were treated as invalid responses and excluded from the analysis.

The qualitative data obtained from these responses were analyzed using thematic content analysis. First, all responses were reviewed thoroughly to gain an overall understanding of the data. Then, similar responses were grouped and categorized based on common themes or patterns. For instance, responses related to health guidance were thematically coded into subcategories such as clothing advice, bathing methods, and dietary recommendations. Physical care-related responses were similarly categorized based on the type of physical care mentioned, including hot compress use, bathing methods, or acupuncture and moxibustion. Coding was conducted manually by the primary researcher, and categorizations were reviewed by a second researcher to ensure consistency and clarity. The frequency of each theme was also counted to understand the prevalence of specific types of care mentioned in the free-text responses.

Ethical consideration

A document was attached to the survey form that described the outline of the study, its purpose, ethical considerations, consent to the study, and the researcher's contact information, and was used to explain the study to the study participants. Research cooperation was voluntary, and the study participants were to return the survey themselves, and answering the survey constituted consent to participating in the study. The questionnaire was anonymous, and no personally identifiable information was collected. Collected data were stored securely and used solely for the purposes of this study. This study was approved by the Medical Research Ethics Committee of the Mie University Hospital (approval number: U2020-004).

## Results

A total of 2,851 questionnaires were distributed (2,482 to hospitals, 200 to clinics, 169 to maternity homes). Responses were received from 1,401 hospital (HP) midwives, 141 clinic (CL) midwives, and 135 maternity home (MH) midwives, totaling 1,677 (response rate: 58.8%).

Experience with health guidance for Hiesho was responded by 1,494 (89.1%), midwives: 1,231 HP midwives (87.9%), 129 CL midwives (91.5%), and 134 MH midwives (99.3%). Experience with treatment was reported by 1,136 (67.7%): 922 HP midwives (65.8%), 93 CL midwives (66.0%), and 121 MH midwives (89.6%). MH midwives had significantly higher response rates for both health guidance and treatment compared to HP and CL midwives (HP: p < 0.001; CL: p <0.001).

**Figure 1:**
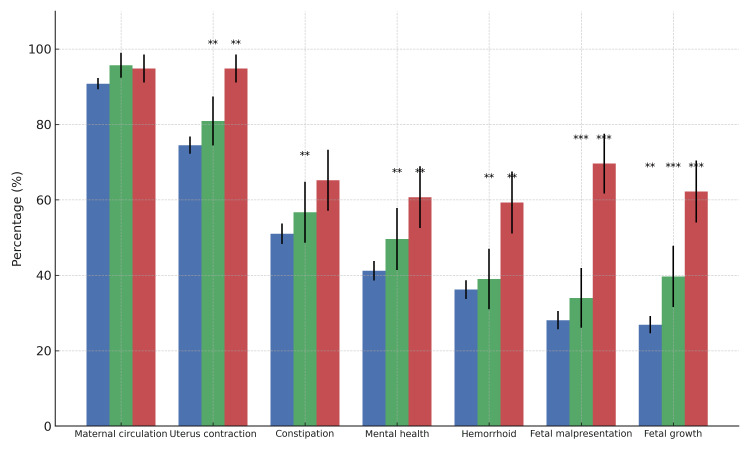
Perceived health effects of Hiesho (cold extremities) during pregnancy among midwives in different facility types The graph shows the percentage of respondents who selected each perceived health effect as associated with Hiesho, by facility type: hospitals (blue), clinics (green), and maternity homes (red). Error bars represent 95% confidence intervals. Significance levels indicate differences between groups (chi-square test with Bonferroni correction): p < .05, **p < .01, ***p < .001

Regarding the effects of Hiesho on pregnancy, the most common response was maternal circulation (1,535 midwives, 91.5%), followed by uterine contractions (1,286 midwives, 76.7%) and constipation (882 midwives, 52.6%). Among those who selected "maternal circulation," there were 1,272 HP midwives (90.8%), 135 CL midwives (95.7%), and 128 MH midwives (94.8%). Although there was a significant difference by facility type (*p*=0.047), post hoc tests revealed no statistically significant differences. Regarding uterine contractions, 1,044 HP midwives (74.5%), 114 CL midwives (80.9%), and 128 MH midwives (94.8%) responded that Hiesho affects them. The response was significantly higher among MH midwives compared to HP and CL midwives (HP: p < 0.001; CL: p = 0.001). For constipation, 714 HP midwives (51.0%), 80 CL midwives (56.7%), and 88 MH midwives (65.2%) selected this response, with the response being significantly higher among MH midwives than HP midwives (p = 0.005). These findings are presented in Figure 1 and Supplementary Table 1.

Pregnant women who midwives considered in need of health guidance, in descending order of frequency, were those wearing light clothing (973 midwives, 65.5%), those experiencing symptoms of Hiesho (925 midwives, 62.2%), and those seeking consultation about Hiesho (810 midwives, 54.5%). There was no significant difference among facility types in the proportion of midwives providing health guidance to pregnant women wearing light clothing (p = 0.525). However, a significant difference was observed regarding guidance for women with symptoms of Hiesho (p = 0.001), with a higher proportion of MH midwives offering such guidance compared to HP midwives (p = 0.001). Similarly, for pregnant women seeking consultation about Hiesho, a significant difference was found among facilities (p = 0.005), with a higher proportion of MH midwives providing guidance compared to both HP (p= 0.005) and CL midwives (p = 0.023). In terms of physical care, the most frequently reported conditions were pregnant women experiencing symptoms of Hiesho (830 midwives, 73.5%), followed by those seeking consultation about Hiesho (547 midwives, 48.4%), and those reporting awareness of uterine contractions (438 midwives, 38.8%). No significant differences were observed among facility types in the proportion of midwives providing physical care to pregnant women with symptoms of Hiesho (p = 0.801) or those seeking consultation about Hiesho (p = 0.070). However, a significant difference was found in the physical care provided to women aware of uterine contractions (p < 0.001), with a higher proportion of MH midwives compared to HP midwives (p = 0.002). These findings are presented in Supplementary Tables 2, 3.

**Figure 2:**
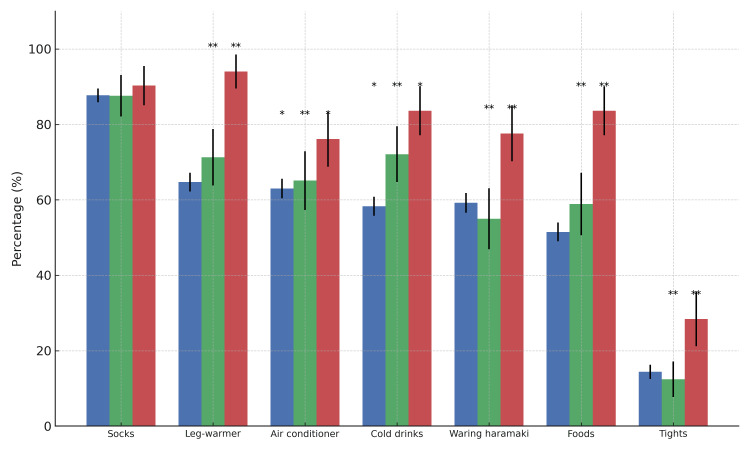
Contents of health guidance for Hiesho (cold extremities) during pregnancy by facility type This figure illustrates the percentage of respondents who provided health guidance on each item related to Hiesho management in pregnant women, categorized by facility type: hospitals (blue), clinics (green), and maternity homes (red). Items include wearing socks, wearing leg warmers, avoiding excessive exposure to air conditioning, avoiding cold drinks, wearing a haramaki (a traditional Japanese abdominal warmer), eating foods that warm the body, and wearing tights. Note: The labels on the x-axis represent abbreviated forms used in the questionnaire and are as follows: “Socks” = “Wearing socks;” “Leg-warmer” = “Wearing leg-warmers;” “Air conditioner” = “Avoiding excessive exposure to the air conditioner;” “Cold drinks” = “Avoiding cold drinks;” “Waring haramaki” = “Wearing haramaki (a traditional Japanese abdominal warmer);” “Foods” = “Eating foods that warm the body;” “Tights” = “Wearing tights.” Error bars represent 95% confidence intervals. Significance levels indicate differences between groups (chi-square test with Bonferroni correction): p < .05, **p < .01

Regarding the specific content of health guidance, the items with the highest responded rates were as follows: wearing socks (1,313 midwives, 87.9%), wearing leg warmers (1,014 midwives, 67.9%), avoiding excessive exposure to air conditioning (961 midwives, 64.3%), avoiding cold drinks (923 midwives, 61.8%), wearing a haramaki (abdomen binder) (904 midwives, 60.5%), eating of food that warm the body (822 midwives, 55.0%), and wearing tights (231 midwives, 15.5%). There was no significant difference among facilities in the response recommending wearing socks (p = 0.705). MH midwives significantly more frequently recommended the use of leg warmers compared to HP and CL midwives (HP: p < 0.001; CL: p < 0.001). Avoiding exposure to too much air conditioning was recommended more by MH midwives than HP midwives (HP midwives: p = 0.007). Avoiding cold drinks followed the order MH midwives > CL midwives > HP midwives, and the differences in responses were significant across each. Wearing haramaki was also more frequently recommended by MH midwives than HP and CL midwives (HP midwives: p < 0.001; CL midwives: p < 0.001). Eating foods that warm the body was more commonly advised by MH midwives than both HP and CL midwives (HP midwives: p< 0.001; CL midwives: p < 0.001). Likewise, recommending the use of tights was significantly more common among MH midwives compared to HP and CL midwives (HP midwives: p < 0.001; CL midwives: p < 0.001). These findings are presented in Figure 2 and Supplementary Table 4.

In the free-text responses, the specific content of health guidance included how to choose clothes (171 responses), avoid exposing neck, wrists, and ankles (164 responses), adjust clothing according to the season (76 responses), avoid exposing abdomen and lower body (59 responses), and adjust the number of layers of clothing (27 responses). Regarding dietary advice, foods that cool the body and should be avoided were mentioned by 176 responses, and foods recommended for warming the body had 166 responses. Foods to avoid included raw leafy vegetables, white sugar, summer foods, and sugary items. Foods to consume commonly included root vegetables and spices such as ginger. Other health guidance included bathing methods (234 responses), exercise methods (70 responses), how to use a hot compress (52 responses), and acupuncture and moxibustion methods (41 responses). 

**Figure 3:**
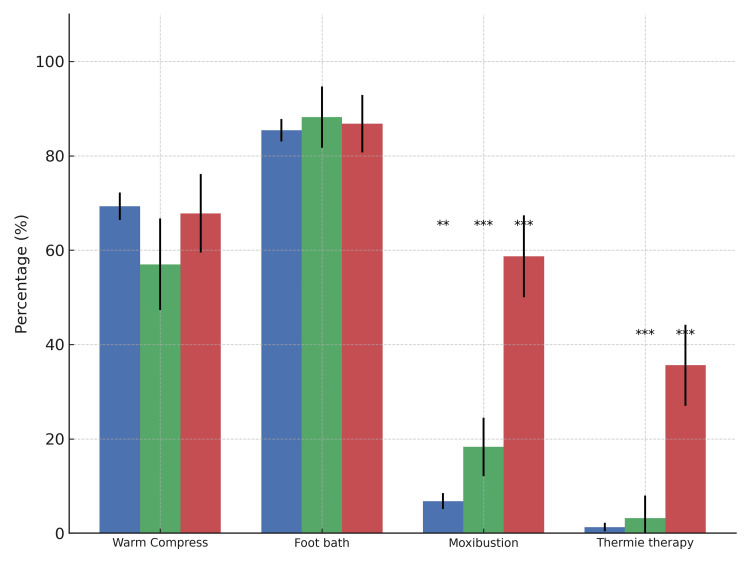
Types of physical care provided for Hiesho (cold extremities) during pregnancy by facility type This figure shows the percentage of respondents who reported providing each type of physical care for pregnant women with Hiesho, categorized by facility type: hospitals (blue), clinics (green), and maternity homes (red). Items include warm compresses, foot baths, moxibustion, and Thermie therapy. Note: The x-axis labels represent shortened forms used in the questionnaire and correspond to the following full descriptions: Error bars represent 95% confidence intervals. Significance levels indicate differences between groups (chi-square test with Bonferroni correction): p < .05, **p < .01, ***p < .001

As for the specific content of physical care, the items with the highest response rates were foot baths (971 midwives, 85.8%), warm compress (771 midwives, 68.1%), moxibustion (150 midwives, 13.1%), and Thermie therapy (58 midwives, 5.1%) (Supplementary Table 5). There were no significant facility differences in the response for foot baths (p = 0.772) or warm compress (p = 0.056). However, there was a significant difference in the use of moxibustion (p < 0.001), with MH midwives practicing it more frequently than HP and CL midwives (HP midwives: p < 0.001, CL midwives: p < 0.001), and CL midwives more than HP midwives (p < 0.001). The use of Thermie therapy also varied significantly by facility (p < 0.001), with MH midwives reporting the highest implementation rates (HP midwives:p < 0.001, CL midwives: p < 0.001). In the free-text responses, 69 responses mentioned performing massage, and 26 responses described specific methods of warm compress therapy. These findings are presented in Figure 3 and Supplementary Table 5.

## Discussion

In this study, a stratified sampling method was used to select study participants, and a nationwide survey was conducted. The 58.8% response rate is acceptable for postal surveys among healthcare professionals, aligning with prior studies reporting average rates around 50-60% for similar populations [7,8]. Among midwives, 89.1% reported providing health guidance on Hiesho, while 67.7% reported performing physical care. The frequency of these practices varied by facility, with midwives working in maternity homes (MH) reporting the highest response rates overall. Previous studies have not reported on the response rates of Hiesho care by midwives, making this study the first to reveal the frequency of Hiesho care by facility type in Japan.

Most responses related to the health effects of Hiesho on pregnancy were negative effects on maternal circulation, with over 90% of midwives from all facility types, and no significant difference among facilities. Thus, Hiesho is believed to be associated with poor maternal circulation, which is shared among Japanese midwives. Previous study suggested that applying cold stimuli (e.g., immersing hands in ice water) in late pregnancy can cause uterine artery constriction and potentially reduce blood flow to the placenta [9]. Therefore, the recognition that Hiesho should be avoided is partially supported by a previous study. Other responses regarding the health effect of Hiesho on pregnancy were significantly more common among MH midwives, suggesting they place greater emphasis on the relationship between Hiesho and pregnancy complications compared to HP midwives or CL midwives.

The most common response about the pregnant women requiring health guidance on Hiesho was those concerned about their light clothing, with no significant difference by facility. This indicates an intention to offer preventative guidance regardless of symptoms, based on the idea that clothing affects body temperature. In contrast, physical care was seen as necessary for pregnant women with symptoms of a cold or uterine contractions and was practiced for alleviating the symptoms. In addition, the response of MH midwives who reported pregnant women experiencing symptoms of uterine contractions or fetal malposition was significantly higher than that of other midwives.

Regarding health guidance, wearing socks among the clothing tips was implemented in more than 80% of all facilities, and there was no significant difference in response rate between facilities. Regarding items other than socks, the implementation rate of MH midwives was significantly higher for all items. MH midwives manage their facilities independently [5] and have enough time to provide health guidance during prenatal checkups.

For physical care, foot bath and warm compress were reported at high response across all types of facilities. Additionally, moxibustion and Thermie therapy were more frequently reported by MH midwives. Foot bath and warm compress have been proven by midwifery and nursing research to have a warming effect on the body and are easy-to-practice care [10,11]. On the other hand, moxibustion, which is mainly practiced in maternity homes, is a traditional treatment method based on traditional Chinese medicine (TCM) [12]. Moxibustion involves burning a herbal preparation containing mugwort to generate heat, which is applied close to the skin until local vasodilation causes hyperemia. The thermal stimulation on the skin surface is believed to also stimulate internal organs [13]. Thermie therapy is a traditional treatment practiced in Japan [14]. It involves rubbing the skin with a rod-shaped device called a Termie-sen, which contains minerals and several types of plants, with the aim of alleviating various symptoms.

Differences in Hiesho care practices by facility type can be understood in light of Japan’s healthcare system. MH midwives only support low-risk pregnancies and are obligated to refer cases with complications to medical institutions [5]. This system necessitates that MH midwives be highly sensitive to subtle changes in maternal health in order to make appropriate care decisions and referrals. HP and CL midwives collaborate with physicians in prenatal care and delivery, requiring some clinical judgment. HP midwives often handle high-risk pregnancies involving medical intervention, while CL midwives focus more on natural births but must still manage complications. In both settings, concern about Hiesho exists, but care is often guided by physician-led treatment plans. These systemic differences may influence the extent to which midwives at each type of facility can implement individualized care, such as Hiesho interventions.

Wearing socks is one of the health guidance that pregnant women can easily follow. Even in the free text section, specific methods were found for avoiding exposure of the abdomen, lower body, neck, wrists, and ankles, and other parts of the body that are attached to the neck, and using clothing to keep warm. In this way, the idea of ​​not cooling the neck and body parts is consistent with the idea of ​​TCM. In TCM, the locations of acupuncture points are indicated, and the acupuncture points on the neck, wrists, and ankles are the points to influence the autonomic nervous system [15]. Based on the above, it is assumed that socks are easy to wear and can cover the ankles, an important part of the body, to prevent coldness. This is the reason the response to health guidance implementation was high. The same reason for wearing leg warmers as socks can be considered. More than half of the midwives provided health guidance about avoiding cold drinks and eating foods that warm the body. Based on the free text of the results, it was suggested that summer food should be avoided. Root vegetables that grow in the soil were thought to have a warming effect on the body. According to the theory of yin and yang in TCM, foods eaten in the summer are thought to have cooling properties [16]. Therefore, we believe that some of the guidance related to eating and drinking is based on the yin-yang theory of TCM.

Based on the above results, practices such as warming the ankles through clothing guidance in health education, applying the yin-yang theory in dietary guidance, and using moxibustion in physical care, were shown to have strong conceptual similarities with TCM. Additionally, Thermie therapy is a traditional Japanese treatment method. Complementary and alternative medicine (CAM) is described as “a broad range of healing philosophies, approaches, and therapies that are not generally used, accepted, studied, understood, or provided by mainstream Western medicine” and includes therapies such as acupuncture and moxibustion, aromatherapy, and herbal preparations [17]. Therefore, most of the Hiesho care methods showed some similarity with concepts for CAM.

The concept of Hiesho and its related care practices in Japan are not unique but show similarities with traditional medical systems in other cultures. In Korean traditional medicine, the concept of "cold syndrome" (Hanjeung) is believed to cause gynecological disorders, including menstrual irregularities and infertility [18]. Similarly, in Unani medicine, individuals with a cold temperament are advised to avoid cold exposure and foods, particularly during pregnancy [19]. These similarities highlight that concern over the impact of cold on reproductive health is common across traditional medical systems.

Pregnant women often experience numerous health issues that may affect normal delivery and fetal development. Even in the absence of complications, many women endure physical discomfort and psychological distress. However, due to the need to be cautious about the use of medications during pregnancy from the perspective of fetal growth and development, there is a demand for safe and effective treatments. For these reasons, many of the responses regarding Hiesho care involved CAM approaches. However, no studies were found that specifically investigated the effectiveness of moxibustion on Hiesho. Although Thermie therapy has been approved by the Ministry of Health, Labour and Welfare as a safe treatment, there have been concerns that long-term use may cause skin conditions [14]. Therefore, in order to improve the quality of Hiesho care for pregnant women, it is necessary to conduct research on the effectiveness of each treatment method.

Limitations of the study and future research directions

This study was a nationwide survey covering hospitals, clinics, and midwifery homes, where most midwives in Japan are employed. Therefore, the findings are considered to reflect the actual practices and perceptions of Hiesho care by midwives across diverse clinical settings. However, several limitations must be acknowledged.

First, this study employed a descriptive, cross-sectional design based on self-reported data. While its primary aim was to explore differences in midwifery care practices across facility types, it does not allow for the identification of causal relationships. As such, any causal interpretations should be avoided, and this limitation should be acknowledged in the discussion.

Second, this study did not investigate how midwives observe and assess Hiesho in pregnant women, how they select specific Hiesho care interventions based on their assessments, or how they evaluate the effectiveness of such care. As a result, the criteria for implementing Hiesho care and the methods for determining its effectiveness remain unclear. Additionally, the reasons why some midwives do not engage in Hiesho care were not explored.

Third, selection bias may have affected the findings. The facilities and midwives that chose to participate may have had a particular interest in Hiesho care, potentially leading to an overestimation of its prevalence and perceived effectiveness. For example, midwives working in midwifery homes, where the number of facilities and staff is limited, reported high rates of care implementation, possibly reflecting a stronger commitment to natural and individualized care practices among those who responded. Furthermore, because this was an anonymous survey, we were unable to collect detailed characteristics of non-responding facilities. This limits our ability to assess differences between respondents and nonrespondents and may introduce nonresponse bias. We acknowledge this limitation and recommend that future studies consider registry-based methods to better address this issue.

Fourth, the use of a self-administered questionnaire introduces the possibility of information bias, including social desirability bias and recall bias. Midwives may have overreported favorable practices or misremembered specific details regarding care implementation. Additionally, variations in the interpretation of the term “Hiesho” or “Hiesho care” may have influenced responses, as these concepts are not yet uniformly defined in clinical practice.

Fifth, the internal consistency of certain questionnaire sections (e.g., health guidance content and specific care practices) was low, suggesting that the items may not have fully captured the intended constructs or that midwives’ understanding of these items varied. This may have led to response variability that affected the reliability of the findings.

Finally, while differences in perceptions and practices were compared across types of facilities, potential confounding factors, such as differences in the clinical risk profiles of pregnant women, staffing levels, and institutional policies, were not controlled for. These contextual variables may partially account for the observed differences and should be considered when interpreting the results. In addition, the analysis did not account for clustering of responses within facilities. Ignoring intraclass correlations may have affected the accuracy of estimated standard errors.

Given these limitations, future research should investigate the clinical decision-making processes midwives use to assess, implement, and evaluate Hiesho care and should incorporate standardized definitions and observational methods to enhance the reliability and validity of findings. Moreover, studies that examine the contextual and organizational factors influencing Hiesho care practices would provide a deeper understanding of implementation across various clinical settings.

## Conclusions

A total of 1,494 midwives (89.1%) reported having experience providing health guidance as Hiesho care, and 1,136 midwives (67.7%) reported having experience providing physical care. Significantly more midwives working in maternity homes had experience with both health guidance and physical care compared to those working in hospitals and clinics. Midwives recognized that Hiesho is believed to be associated with poor maternal circulation in pregnant women. The Hiesho care methods showed some similarity with concepts for CAM. Further research is needed to examine the evidence and effectiveness of the Hiesho care practices that have been implemented thus far.

## Appendices

**Table 1:** Health effect of Hiesho (cold extermities) on pregnancy HP: midwife working in hospital; CL: midwife working in clinic; MH: midwife working in maternity home Indicates the number (percentage) of midwives who consider the symptoms to be affected by Hiesho. Chi-square test was used to test for differences in percentages between working facilities. a: For significant differences, the results of post hoc tests using Holm's method are shown

	HP (n = 1401)	CL (n = 141)	MH (n = 135)	Chi-square value	p-value	Post hoc test^ a^
Maternal circulation	90.8% (CI: 89.3-92.3)	95.7% (CI: 92.4-99.1)	94.8% (CI: 91.1-98.6)	6.09	0.047	HP vs CL: 0.176, HP vs MH: 0.303, CL vs MH: 0.782
Uterus contraction	74.5% (CI: 72.2-76.8)	80.9% (CI: 74.4-87.3)	94.8% (CI: 91.1-98.6)	28.04	<0.001	HP vs CL: 0.103, HP vs MH: <0.001, CL vs MH: 0.001
Constipation	51.0% (CI: 48.3-53.6)	56.7% (CI: 48.6-64.9)	65.2% (CI: 57.1-73.2)	11.05	0.004	HP vs CL: 0.350, HP vs MH: 0.005, CL vs MH: 0.350
Mental health	41.2% (CI: 38.6-43.8)	49.6% (CI: 41.4-57.9)	60.7% (CI: 52.5-69.0)	21.55	<0.001	HP vs CL: 0.120, HP vs MH: <0.001, CL vs MH: <0.120
Hemorrhoid	36.2% (CI: 33.7-38.7)	39.0% (CI: 31.0-47.1)	59.3% (CI: 51.0-67.5)	27.8	<0.001	HP vs CL: 0.521, HP vs MH: <0.001, CL vs MH: 0.002
Fetal malpresentation	28.1% (CI: 25.7-30.4)	34.0% (CI: 26.2-41.9)	69.6% (CI: 61.9-77.4)	98.31	<0.001	HP vs CL: 0.143, HP vs MH: <0.001, CL vs MH: <0.001
Fetal growth	26.9% (CI: 24.6-29.2)	39.7% (CI: 31.6-47.8)	62.2% (CI: 54.0-70.4)	77.77	<0.001	HP vs CL: 0.002, HP vs MH: <0.001, CL vs MH: <0.001

**Table 2:** Pregnant women who are considered by midwives required health guidance for Hiesho care (care for cold extremities) HP: midwife working in hospital; CL: midwife working in clinic; MH: midwife working in maternity home Indicates the number (percentage) of midwives who think that these conditions require Hiesho care for pregnant women. Chi-square test was used to test for differences in percentages between working facilities. a: For significant differences, the results of post hoc tests using Holm's method are shown

	HP (n = 917)	CL (n = 93)	MH (n = 120)	Chi-square value	p-value	Post hoc test^a^			
Symptoms of Hiesho	73.3% ( CI: 70.3-76.0)	76.3% (CI: 66.8–83.8)	72.5% (CI: 63.9–79.7)	0.47	0.801				
Seeking consultation about Hiesho	47.3% (CI: 44.1-50.6)	46.2% ( CI: 36.5-56.3)	58.3% (CI: 49.4-66.8)	5.34	0.07				
Awareness of uterine contractions	36.4% (CI: 33.4-39.6)	39.8% (CI: 30.4-49.9)	55.8% (CI: 46.9-64.4)	16.89	<0.001	HP vs CL: 0.573, HP vs MH: 0.002, CL vs MH: 0.054			
All pregnant women	21.4% CI: 18.8-24.1)	24.7% (CI: 17.1-34.4)	55.8% (CI: 46.9-64.4)	66.67	<0.001	HP vs CL: 0.432, HP vs MH: <0.001, CL vs MH: <0.001			
Fetal malpresentation	8.8% (CI: 7.2-10.8)	21.5% ( CI: 14.4-30.9)	50.8% (CI: 42.0-59.6)	156.66	<0.001	HP vs CL: <0.001, HP vs MH: <0.001, CL vs MH: <0.001			

**Table 3:** Pregnant women who are considered by midwives required treatment for Hiesho care (care for extremities) HP: midwife working in hospital; CL: midwife working in clinic; MH: midwife working in maternity home Indicates the number (percentage) of midwives who think that these conditions require Hiesho care for pregnant women. Chi-square test was used to test for differences in percentages between working facilities. a: For significant differences, the results of post hoc tests using the Holm's method are shown

	HP (n = 917)	CL (n = 93)	MH (n = 120)	Chi-square value	p-value	Post hoc test^a^
Symptoms of Hiesho	73.3% (CI: 70.3-76.1)	76.3% (CI: 66.8-83.7)	72.5% (CL: 63.7-80.0)	0.47	0.801	
Seeking consultation about Hiesho	47.3% (CL: 44.1-50.6)	46.2% (CL: 36.5-56.2)	58.3% (CL: 49.2-66.9)	5.34	0.07	
Awareness of uterine contractions	36.4% (CL: 33.4-39.6)	39.8% (CL: 30.6-49.9)	55.8% (CL: 46.8-64.4)	16.89	<0.001	HP vs CL: 0.573, HP vs MH: 0.002, CL vs MH: 0.054
All pregnant women	21.4% (CL: 18.9-24.2)	24.7% (CL: 17.0-34.5)	55.8% (CL: 46.8-64.4)	66.67	<0.001	HP vs CL: 0.432, HP vs MH: <0.001, CL vs MH: <0.001
Fetal malpresentation	8.8% (CL: 7.1-10.9)	21.5% (CL: 14.6-30.6)	50.8% (CL: 41.7-59.9)	156.66	<0.001	HP vs CL: <0.001, HP vs MH: <0.001, CL vs MH: <0.001

**Table 4:** Practical details of health guidance as Hiesho care (care for cold extremities) HP: midwife working in hospital; CL: midwife working in clinic; MH: midwife working in maternity home Indicates the number (percentage) of midwives who perform Hiesho care, both for health guidance and physical care for pregnant women. Chi-square test was used to test for differences in percentages between working facilities. a: For significant differences, the results of post hoc tests using Holm's method are shown

	HP (n = 1419)	CL (n = 141)	MH (n = 135)	Chi-square value	p-value	Post hoc test^a^
Wearing socks	87.7% (CI: 85.9-89.4)	87.6% (CI: 81.2–92.2)	90.3% (CI: 83.9-94.4)	0.81	0.705	
Wearing leg-warmer	64.7% (CI: 62.2-67.2)	71.3% (CI: 63.2–78.3)	94.0% (CI: 88.1-97.1)	48.56	<0.001	HP vs CL: 0.145, HP vs MH: <0.001, CL vs MH: <0.001
Avoiding too much exposure to the air conditioner	63.0% (CI: 60.4-65.5)	65.1% (CI: 56.8-72.5)	76.1% (CI: 68.0-82.6)	9.16	0.009	HP vs CL: 0.005, HP vs MH: <0.001, CL vs MH: 0.026
Avoiding cold drinks	58.3% (CI: 55.7-60.8)	72.1% (CI: 64.0-78.9)	83.6% (CI: 76.1-89.1)	39	<0.001	HP vs CL: 0.005, HP vs MH: <0.001, CL vs MH: 0.026
Waring haramaki (abdomen binder)	59.2% (CI: 56.6-61.7)	55.0% (CI: 46.7-63.0)	77.6% (CI: 69.4-84.2)	18.87	<0.001	HP vs CL: 0.398, HP vs MH: <0.001, CL vs MH: <0.001
Eating foods that warm the body	51.5% (CI: 48.9-54.0)	58.9% (CI: 50.6-66.7)	83.6% (CI: 76.1-89.1)	51.12	<0.001	HP vs CL: 0.116, HP vs MH: <0.001, CL vs MH: <0.001
Wearing tights	14.4% (CI: 12.6-16.4)	12.4% (CI: 7.7-19.2)	28.4% (CI: 21.2-36.6)	19.08	<0.001	HP vs CL: 0.598, HP vs MH: <0.001, CL vs MH: <0.001

**Table 5:** Practical details of treatment as Hiesho care (care for cold extremities) HP: midwife working in hospital; CL: midwife working in clinic; MH: midwife working in maternity home Indicates the number (percentage) of midwives who perform Hiesho care, both for health guidance and physical care for pregnant women. Chi-square test was used to test for differences in percentages between working facilities. a: For significant differences, the results of post hoc tests using Holm's method are shown. b: Fisher's exact test was performed

	HP (n = 918)	CL (n = 93)	MH (n = 121)	Chi-square value	p-value	Post hoc test^a^
Warm compress	69.3% (CI: 66.3-72.1)	57.0% (CI: 46.9-66.6)	67.8% (CI: 59.0-75.5)	5.88	0.056	
Foot bath	85.4% (CI: 83.0-87.6)	88.2% (CI: 80.3-93.3)	86.8% (CI: 79.6-91.8)	0.64	0.772	
Moxibustion	6.8% (CI: 5.3-8.7)	18.3% (CI: 12.1-26.8)	58.7% (CI: 49.7-67.1)	252.98	<0.001	HP vs CL: 0.004, HP vs MH: <0.001, CL vs MH: <0.001
Thermie therapy	1.3% (CI: 0.7-2.4)	3.2% (CI: 1.1-9.0)	35.6% (CI: 27.5-44.6)	209.23	<.0001^b^	HP vs CL: 0.153, HP vs MH: <0.001, CL vs MH: <0.001

Supplementary file: questionnaire

"National Survey on Hiesho Care for Pregnant Women and Pregnancy Outcomes"

Purpose of the questionnaire: This survey focuses on the following four main topics:

1.           Your views on the relationship between pregnancy and Hiesho.

2.           Your experience providing health guidance to pregnant women.

3.           Your experience providing thermal care to pregnant women.

4.           Your professional experience as a midwife.

Please respond based on your experience specifically with pregnant women, not postpartum or laboring women.

After reading the explanation titled “Request for Participation in the National Survey on Hiesho Care for Pregnant Women and Pregnancy Outcomes,” please check the box below to indicate your consent before proceeding to the questionnaire.

                                                           □ I agree to participate in this study.

Instructions for responding:

·        Estimated time to complete approx. 10 minutes.

·        Please answer based only on your personal experience as a midwife.

·        The questionnaire consists of three pages.

·        We would appreciate it if you could respond and return the questionnaire within two weeks of receipt.

·        Please place only the completed questionnaire in the return envelope and drop it in the nearest mailbox.

◆Please answer your thoughts on pregnancy and Hiesho.

【1】Have you ever heard the belief that “pregnant women should not get cold” or “should stay warm”? (Select one.)

　　　　1. Yes

　　　　2. No

【2】Where did you hear about this belief? (Multiple answers allowed.)

1.   School lectures or classes　　　　　　

2.   School clinical training　

3.   Books, journal articles, or magazines　

4.   Workshops or academic conferences by healthcare professionals

5.   From other healthcare professionals (doctors, nurses, midwives)　　

6.   Observed in clinical settings

7.   From nonhealthcare individuals (please specify):　　　　　            　　 　　　　)

8.   Other (please specify):　　　                                      　　 　　　　)

【3】Do you personally believe that “pregnant women should avoid getting cold/stay warm”? (Select one.)

　　　　1. Yes

　　　　2. No

【4】Please explain your reason

【5】What effect do you think Hie has on pregnant women and pregnancy?

(Circle the number that applies; multiple answers allowed.)

1.    Uterine contractions   

2.    Abnormal fetal positions   

3.    Constipation    

4.    Hemorrhoids

5.    Fetal growth and development     

6.    Mental health

7.    Maternal circulation

8.    Others (please specify):　　　　　　　　 　　　　

◆Please answer about your experience in providing health guidance to pregnant women.

【6】Have you ever provided health guidance to pregnant women aimed at preventing or alleviating Hie? (Select one.)

1.   Yes 　　　　  Proceed to Q7, Q8, Q9

2.   No　　　　    Proceed to Q10

【7】What kind of health guidance did you provide to pregnant women?

(Circle the number that applies; multiple answers allowed.)

1.   Wearing socks　　

2.   Wearing leg warmers　　

3.   Wearing tights　　

4.   Wearing Haramaki (abdomen binder)

5.   Avoiding cold drinks

6.   Eating foods that warm the body　　

7.   Avoiding exposure to too much air conditioning

8.   Others (please specify):　                                 　　　　 　　　　

【8】What kind of pregnant women were provided health guidance regarding Hie?

(Circle the applicable number; multiple answers possible.)

1.   All pregnant women　　　

2.   Having symptoms of Hiesho

3.   Awareness of uterine contractions

4.   Fetal malpresentation　　

5.   Seeking consultation about Hiesho　　

6.   Wearing light clothing

7.   Others (please specify):　　　　　　　 　                                　　　

【9.1】Do you provide health guidance for Hiesho according to the number of periods of pregnancy?

      (If you selected 1, please also check one of A through C.)

1.   Yes     　　　A. First trimester　B. Second trimester　　C. Third trimester　

2.   No

【9.2】Do you provide health guidance for Hiesho according to the season? (If you selected 1, please also check one of D through G.)

1.   Yes           D. Spring　　E. Summer　　F. Fall　　G. Winter

2.   No

◆Please answer about your experience in providing treatment for pregnant women.

【10】Have you ever provided treatment to pregnant women aimed at preventing or alleviating Hie? (Select one.)

1.   Yes            Proceed to Q11, Q12, Q13

2.   No             Proceed to Q14

【11】What kind of treatment did you provide? (Circle all applicable options.)

1.   Warm compress

2.   Foot bath

3.   Thermie

4.   Moxibustion

5.   Others (please specify):　　　                              　　　　 　 　　　　

【12】What kind of pregnant women were provided treatment regarding Hie?

(Circle the applicable number; multiple answers possible.)

1.   All pregnant women　　　

2.   Having symptoms of Hiesho

3.   Awareness of uterine contractions

4.   Fetal malpresentation　　

5.   Seeking consultation about Hiesho　　

6.   Others (please specify:　　                              　　　　　　　　　　

【13.1】Do you provide treatment for Hiesho according to the number of periods of pregnancy?

        (If you selected 1, please also check one of A through C.)

1.   Yes　　　     A. First trimester　B. Second trimester　　C. Third trimester

2.   No

【13.2】Do you provide treatment for Hiesho according to the season?

     (If you selected 1, please also check one of D through G.)

1.   Yes           D. Spring　　E. Summer　　F. Fall　　G. Winter

2.   No

Please answer questions about your experience as a midwife.

【14.1】Please answer the number of years you have been practicing as a midwife.

【14.2】Please answer the type of work facility by number (select and circle one from 1 to 8).

1.   Health center

2.   Prefecture/municipality

3.   Hospital (20 or more beds)

4.   Clinic (with beds)

5.   Clinic (without beds)

6.   Maternity home

7.   Nursing school/research institute

8.   Others (please specify):　　                               　　　　　 　　　　

Thank you for your cooperation. This concludes this survey. We apologize for the inconvenience, but please enclose only the questionnaire form in the return envelope and mail it to your local mailbox.
